# Bidirectional Relationship Between Subjective Age and Fear of Falling: Findings From the NHATS

**DOI:** 10.1093/geroni/igaa057.754

**Published:** 2020-12-16

**Authors:** Yuxiao Li, Minhui Liu, Christina E Miyawaki, Tianxue Hou, Xiaocao Sun, Siyuan Tang, Sarah Szanton

**Affiliations:** 1 Central South University, Changsha, Hunan, China; 2 Central South University, Baltimore, Maryland, United States; 3 University of Houston, Seattle, Washington, United States; 4 Central South University, Changsha City, Hunan, China; 5 Johns Hopkins University, Baltimore, Maryland, United States

## Abstract

Subjective age refers to how young or old people experience themselves to be. It is associated with health status, physical function, and mental processes that influence the occurrence of fear of falling, which is a severe problem in older adults. The present study aims to contribute to existing knowledge by examining the relationships between subjective age and fear of falling. Participants were community-dwelling adults aged 65 years and older drawn from the 2011 (T1) - 2015 (T5) waves of the National Health and Aging Trends Study. Subjective age was measured by asking, “What age do you feel most of the time?” Fear of falling was measured with the question: “In the last month, did you worry about falling down?” The analyses included 1,984 participants without fear of falling at baseline, on average, 74.9±6.6 years old, female (53%), and non-Hispanic whites (76%). Eighty percent of the participants felt younger, and 4% felt older than their chronological age, but 21% of them had a fear of falling. Participants who experienced fear of falling at T5 tended to be female, non-Hispanic whites, and live alone. Generalized estimating equations revealed that an “older” subjective age independently predicted fear of falling (OR, 95%CI= 1.02, 1.01-1.02) controlling for demographics and health conditions. Fear of falling predicted an “older” subjective age (OR, 95%CI= 3.38, 1.72-6.66) after adjustment. These findings indicated bidirectional relationships between subjective age and fear of falling. Subjective age may help identify individuals who are at risk for fear of falling in older adults.

